# Intrauterine Growth Restriction Followed by Oxygen Support Uniquely Interferes with Genetic Regulators of Myelination

**DOI:** 10.1523/ENEURO.0263-20.2021

**Published:** 2021-07-02

**Authors:** Jill Chang, Abhineet Sharma, Mirrah Bashir, Camille M. Fung, Robert W. Dettman, Maria L. V. Dizon

**Affiliations:** 1 Department of Pediatrics, Division of Neonatology, Ann & Robert H. Lurie Children’s Hospital of Chicago, Chicago, IL 60611; 2 Department of Pediatrics, University of Utah, Salt Lake City, UT 84112

**Keywords:** cerebral palsy, oligodendroglial, perinatal brain injury, RNA sequencing, white matter injury

## Abstract

Intrauterine growth restriction (IUGR) and oxygen exposure in isolation and combination adversely affect the developing brain, putting infants at risk for neurodevelopmental disability including cerebral palsy (CP). Rodent models of IUGR and postnatal hyperoxia have demonstrated oligodendroglial (OL) injury with subsequent white matter injury (WMI) and motor dysfunction. Here, we investigate transcriptomic dysregulation in IUGR with and without hyperoxia exposure to account for the abnormal brain structure and function previously documented. We performed RNA sequencing and analysis using a mouse model of IUGR and found that IUGR, hyperoxia, and the combination of IUGR with hyperoxia (IUGR/hyperoxia) produced distinct changes in gene expression. IUGR in isolation demonstrated the fewest differentially expressed genes (DEGs) compared with control. In contrast, we detected several gene alterations in IUGR/hyperoxia; genes involved in myelination were strikingly downregulated. We also identified changes to specific regulators including TCF7L2, BDNF, SOX2, and DGCR8, through ingenuity pathway analysis (IPA), that may contribute to impaired myelination in IUGR/hyperoxia. Our findings show that IUGR with hyperoxia induces unique transcriptional changes in the developing brain. These indicate mechanisms for increased risk for WMI in IUGR infants exposed to oxygen and suggest potential therapeutic targets to improve motor outcomes.

## Significance Statement

This study demonstrates that perinatal exposures of intrauterine growth restriction (IUGR) and/or postnatal hyperoxia result in distinct transcriptomic changes in the developing brain. In particular, we found that genes involved in normal developmental myelination, myelin maintenance, and remyelination were most dysregulated when IUGR was combined with hyperoxia. Understanding how multiple risk factors lead to white matter injury (WMI) is the first step in developing future therapeutic interventions. Additionally, because oxygen exposure is often unavoidable after birth, an understanding of gene perturbations in this setting will increase our awareness of the need for tight control of oxygen use to minimize future motor disability.

## Introduction

White matter injury (WMI) following *in utero* hypoxic-ischemic (HI) events, stroke and prematurity is well documented (Volpe et al., 2011; Back, 2015; van Tilborg et al., 2018). The WMI that occurs secondary to these disease processes puts neonates at greater risk of developing motor dysfunction including cerebral palsy (CP; Silbereis et al., 2010). There remains, however, a large percentage of neonates that develop CP who have no history of one of these identified perinatal incidents. This gap in knowledge makes it difficult to develop therapeutic interventions for what often results in lifelong disability.

Intrauterine growth restriction (IUGR) is defined as a significant reduction in fetal growth resulting in birth weight <10th percentile for gestational age (Battaglia and Lubchenco, 1967; Schroder, 2003). It affects ∼5% of pregnancies worldwide (Mandruzzato et al., 2008; Tolcos et al., 2011) and results in an increased risk of mortality and significant morbidities. A number of population-based cohort studies have shown a 5- to 7-fold increased risk of developing CP in growth restricted infants (Ahlin et al., 2013; Dahlseng et al., 2014; Blair and Nelson, 2015; Freire et al., 2015; Streja et al., 2015; Mor et al., 2016). Existing animal studies of IUGR demonstrate evidence of oligodendroglial (OL) injury and subsequent WMI (Olivier et al., 2007; Tolcos et al., 2011; Volpe et al., 2011; Reid et al., 2012; Basilious et al., 2015; Rideau Batista Novais et al., 2016), specifically, a decrease in immature/premyelinating OLs and total OL population (Chang et al., 2018). This is similar to findings in models of prematurity and HI injury where blocked maturation of oligodendrocyte progenitor cells (OPCs) is implicated in impaired white matter development (Chang et al., 2018). Despite these cellular changes, the molecular mechanisms remain incompletely understood. Alterations in gene expression and signaling pathways that lead to abnormal white matter development in the developing brain are under investigation (Hedtjarn et al., 2004; Guedj et al., 2015; Rideau Batista Novais et al., 2016).

Interestingly, not all IUGR infants go on to develop CP (Ahlin et al., 2013; Dahlseng et al., 2014; Blair and Nelson, 2015; Freire et al., 2015; Streja et al., 2015; Mor et al., 2016), suggesting that additional unidentified factors are involved in the WMI that occurs in this population. It is well documented that, in addition to neurodevelopmental impairment, growth restricted infants are at an increased risk of cardiovascular and pulmonary morbidities, including bronchopulmonary dysplasia and pulmonary hypertension (Rozance et al., 2011; Eriksson et al., 2014; Mestan et al., 2014). To provide appropriate support of their cardiorespiratory status, these infants are often admitted to the neonatal intensive care unit and exposed to supraphysiologic oxygen. Rodent studies have found that hyperoxia exposure alone results in damage to the developing white matter including ultrastructural changes in myelin, decreased total OLs, and decreased myelin proteins (Gerstner et al., 2008; Schmitz et al., 2011; Ramani et al., 2013; Ritter et al., 2013). Perinatal brain injury is likely the result of multiple exposures during a critical neurodevelopmental window (Schmitz et al., 2011; Basilious et al., 2015). A multihit hypothesis would provide an explanation for why some IUGR infants go on to develop more severe motor dysfunction and CP. Understanding how multiple risk factors affect the developing brain leading to WMI can lead to modifiable clinical approaches in this high-risk population.

Recently, the multihit hypothesis was tested in a mouse model of IUGR, with and without supplemental oxygen (Chang et al., 2018). Postnatal hyperoxia exposure independently resulted in white matter dysfunction different from that seen in IUGR. IUGR demonstrated changes in OL populations and myelin thickness, while hyperoxia resulted in impaired myelin integrity and decreased white matter tract volume on MRI, suggesting a different mechanism of injury between these exposures. Additionally, a persistent and more complex type of WMI was seen with the combined insult of IUGR with hyperoxia. In contrast to transient changes in OL populations in IUGR, IUGR with hyperoxia resulted in sustained OL alterations into adulthood. The combination of IUGR and hyperoxia also led to more pronounced WMI on MRI and gait changes in adult mice (Chang et al., 2018). The different findings seen with these perinatal exposures support a multihit hypothesis for WMI and also highlight that different mechanisms may be involved with each exposure.

Here, we evaluated the multihit injury model using RNA sequencing (RNA-seq), to test the hypothesis that IUGR and postnatal hyperoxia alter distinct gene networks involved in brain development. We found that IUGR and hyperoxia alone did, indeed, result in distinct changes in gene expression. We also observed that the combination of IUGR with hyperoxia (IUGR/hyperoxia), compared with either condition in isolation, specifically affected genes related to myelination. ingenuity pathway analysis (IPA) identified significant dysregulation of the Wnt/β-catenin signaling pathway through TCF7L2 in both hyperoxia and IUGR/hyperoxia. Additionally, IPA identified dysregulation of specific regulators BDNF, SOX2, and DGCR8 that may be responsible for the impaired myelination in IUGR/hyperoxia. These findings demonstrate that different perinatal exposures result in distinct transcriptomic changes in the developing brain, and further support our multihit hypothesis that exposure of growth restricted infants to therapeutic oxygen results in WMI and potential development of CP.

## Materials and Methods

### Animals

Wild-type C57BL/6 mice were purchased from Charles River. All mice were housed in a facility with a 12/12 h light/dark cycle and allowed access to food and water *ad libitum*. Experiments were conducted according to protocols approved by the Institutional Animal Care and Use Committee and Northwestern Center for Comparative Medicine. Animal procedures were conducted in accordance with the National Institutes of Health *Guide for the Care and Use of Laboratory Animals*.

### Murine IUGR model

Uteroplacental insufficiency is the most common cause of IUGR in developed countries (Fung et al., 2011). Thromboxane A_2_ (TXA_2_), is a vasoconstrictor overly expressed in mothers whose pregnancies are complicated by hypertension, cigarette smoking, and poorly controlled diabetes (McAdam et al., 2005; Hayakawa et al., 2006; Fung et al., 2011; Gibbins et al., 2018). Infusion of TXA_2_-analog U-46619 has been demonstrated to result in placental vasculature reduction, suggesting placental vascular insufficiency, similar to human placental pathology resulting in IUGR (Gibbins et al., 2018). This model does not require invasive surgery, and it is physiologically relevant to human IUGR pregnancies.

Micro-osmotic Alzet pumps (model 1007D, 0.5 ml/h; DURECT Corporation) were implanted into gravid mice at 12.5 d after coitus, correlating with the last trimester of mouse pregnancy. Pumps were inserted into a subcutaneous pocket created in the hip space. The pumps contained either the TXA_2_-analog U-46619 (Cayman Chemical) dissolved in 0.5% ethanol or 0.5% ethanol (vehicle) which was continuously infused at 2000 ng/h throughout the remainder of pregnancy (Fung et al., 2011). Previous model characterization has shown that plasma 11-dehydrothromboxane B_2_ levels were similar between the vehicle and U-46619 exposed fetuses, providing evidence that U-46619 did not cross the placenta to affect the pups directly (Fung et al., 2011). Following spontaneous delivery, pups were weighed on postnatal day (P)0. Pups born to dams receiving TXA_2_-analog and weighing <1.266 g, <10th percentile for weight based on sham pup weights, were assigned to the IUGR group. Using this cutoff, approximately one-third of TXA_2_-analog pups were defined as small for gestational age (SGA), which is similar to the incidence of human SGA infants, born to mothers with uteroplacental insufficiency in IUGR epidemiological studies (Eskenazi et al., 1993). Pups born to dams receiving 0.5% ethanol and weighing >1.266 g (>10th percentile) were assigned to the vehicle group. All pups were cross-fostered to unmanipulated mouse dams postdelivery to minimize the surgical effects of pump insertion in the birth dams.

### Postnatal hyperoxia exposure

Litters of vehicle and IUGR pups were placed in either 75% oxygen (hyperoxia) in a Plexiglas chamber (Biospherix) or 21% oxygen (room air) within 24 h after birth for 14 d (Aslam et al., 2009; Lee et al., 2014). Exposure to hyperoxia was continuous, with brief interruption only for animal care (<10 min/d). The concentration of oxygen was maintained with an oxygen controller (ProOx, Biospherix). Ventilation within the chamber was adjusted to remove CO_2_ such that it did not exceed 0.5%. A hygro-thermometer was used in the chamber to monitor temperature and humidity. Temperature in the chamber did not exceed 23°C and humidity level was maintained using dishes of desiccant in the bottom of the chamber. A foster dam was placed in the hyperoxia chamber with each vehicle or IUGR litter, and foster dams were rotated from hyperoxia to room air every 24–48 h to prevent excessive oxygen toxicity to the adult animals. Litters were removed from the hyperoxia chamber at 14 d and euthanized for tissue collection.

### RNA sequencing, analysis, and bioinformatics

P14 pups were decapitated, whole brains were removed, and right hemispheres were immediately placed in RNAlater solution (Invitrogen) and stored at −80°C until RNA extraction. Total RNA was isolated using the mirVana MiRNA Isolation kit (Thermo Fisher Scientific) per manufacturer protocol and quantified with NanoDropTM Spectrophotometer (Thermo Fisher Scientific). Samples were submitted to Northwestern University Feinberg School of Medicine NUSeq Core facility for TruSeq stranded mRNA sequencing library preparation and HiSeq high throughput sequencing using the Illumina platform (Illumina). For pooled samples, two samples were submitted for each of the four experimental groups, making a total of eight samples submitted for RNA sequencing. Pooled RNA samples consisted of a mix of sex with two to three animals per sample. For individual, non-pooled, RNA-seq, sample sizes were as follows: vehicle/normoxia (control) *n* = 7 (four females, three males), IUGR/normoxia *n* = 5 (one female, four males), vehicle/hyperoxia *n* = 7 (two females, five males), IUGR/hyperoxia *n* = 8 (three females, five males). To assess purity, each RNA sample underwent Bioanalyzer analysis; an RNA Integrity Number score of 7 or higher was used to indicate sufficient quality to proceed with library construction. The RNA samples were enriched for mature RNA and fragmented to obtain RNA fragments ∼50 bp in size. A cDNA library was prepared with adapters added for paired end sequencing on the Illumina platform.

Sequence analysis and bioinformatics was conducted by the Advanced Bioinformatics and Bio-Computation Core Facility at the Center for Genetic Medicine at Northwestern University. The quality of DNA reads, in fastq format, was evaluated using FastQC (Babraham Bioinformatics, Babraham Institute, Cambridge, UK). Adapters were trimmed and reads of poor quality or aligning to rRNA sequences were filtered. The cleaned reads were aligned to the *Mus musculus* genome (mm10) using STAR (Dobin et al., 2013) and Ceto (https://github.com/ebartom/NGSbartom). Read counts for each gene were calculated using HTSeq-Counts (Anders et al., 2014) in conjunction with a gene annotation file for mm10 obtained from UCSC (University of California Santa Cruz; http://genome.ucsc.edu). A comprehensive QC report was generated using MultiQC (Ewels et al., 2016). Differential expression was determined using DESeq2 (Love et al., 2014). The cutoff for determining significantly differentially expressed genes (DEGs) was a false discovery rate (FDR)-adjusted *p* < 0.05.

For subanalysis, gene expression results were also categorized by brain cell type using a transcriptome database created by the Barres lab at Stanford University (Zhang et al., 2014). RNA sequencing of purified neurons, astrocytes, microglia, endothelial cells, pericytes, and various maturation states of oligodendrocytes from mouse cortex were used to generate this high-resolution transcriptome database of >22,000 genes.

Co-expression analysis was performed using the Co-Expression Molecules identification Tool (CEMiTool) package in Bioconductor using variance stabilizing transformation and an FDR cutoff of 0.05 (Russo et al., 2018). IPA (QIAGEN) was used to identify significant biological pathways from the RNA-seq datasets (Krämer et al., 2014). A list of detected genes and detected proteins was used as the data input, using a q < 0.05 cutoff for the gene pathway and *p* < 0.1 cutoff for the protein pathway analyses, such that only significant genes/proteins were considered for significant pathways. The “user dataset” option was chosen to use each individual detected gene/protein data set as the “reference set” for which to generate significant pathways. Pathways from the “diseases and biological functions” category were used for comparison analyses. Fisher’s *t* test of *p* < 0.05 was used to determine statistical significance of a pathway.

### Validation of gene expression

To validate RNA-seq results, qRT-PCR was performed on five genes related to myelination that were found by RNA-seq to be significantly changed. Total RNA from the right hemisphere at P14 was isolated and quantified as described above: vehicle/normoxia (control): *n* = 15 (seven females, eight males), IUGR/normoxia: *n* = 10 (four female, six males), vehicle/hyperoxia: *n* = 12 (five females, seven males), IUGR/hyperoxia: *n* = 14 (seven females, seven males). cDNA was prepared using the TaqMan Advanced miRNA cDNA Synthesis kit (Applied Biosystems). The following Taqman Advanced 20× Assays were used: *Mobp* (mm02745649_m1), *Mbp* (mm01266402_m1), *Cnp* (mm01306640_m1), *Mog* (mm00447824_m1), and *Plp1* (mm01297210_m1), and *Gapdh* (mm99999915_g1; Applied Biosystems) as loading control. Real-time PCR was performed using Bio-Rad CFX Real Time PCR Detection System and Software (Bio-Rad Laboratories). Technical replicates of four were used for each sample. Relative gene target amounts were normalized to the housekeeping gene *Gapdh* using the ΔΔCT method (Livak and Schmittgen, 2001). Regression was used to compare estimates of fold difference between RNA-seq and validation using Prism, version 7.0 (GraphPad Software Inc).

### Region of interest analysis

Tissue from the following four brain regions were collected for analysis. Corpus callosum: control: *n* = 7 (two female, five male), IUGR/normoxia: *n* = 9 (four female, five male), vehicle/hyperoxia: *n* = 8 (four female, four male), IUGR/hyperoxia: *n* = 6 (four female, one male); internal capsule: control: *n* = 6 (two female, four male), IUGR/normoxia: *n* = 8 (three female, five male), vehicle/hyperoxia: *n* = 8 (four female, four male), IUGR/hyperoxia: *n* = 6 (three female, three male); subcortical white matter (SCWM): control: *n* = 5 (two female, three male), IUGR/normoxia: *n* = 4 (two female, two male), vehicle/hyperoxia: *n* = 4 (1 female, three male), IUGR/hyperoxia: *n* = 6 (five female, one male); and cerebellum: control: *n* = 4 (four male), IUGR/normoxia: *n* = 7 (two female, five male), vehicle/hyperoxia: *n* = 7 (three females, four males), IUGR/hyperoxia group: *n* = 6 (three female, three male). Mice were deeply anesthetized, then decapitated, brains were removed and chilled in ice-cold RNAlater solution (Invitrogen) for 5 min, cut into 1-mm sections using a stainless-steel mouse brain slicer, then white matter regions of interest were dissected using tungsten needles. Dissected region of interest tissue was placed in RNAlater and stored at −80°C until RNA extraction. Total RNA isolation, cDNA synthesis, and quantitative RT-PCR were performed as described above. Taqman Advanced 20× Assays for *Mobp*, *Mbp*, *Cnp*, *Mog*, *Plp1*, and *Gapdh* were used as for entire hemisphere above.

### Statistical analysis

Four to fifteen mice were used per treatment group. Mice from both sexes were used. In order to account for the intrauterine environment as a cofounder in developmental studies, mice were taken from at least two independent litters. The comparative C_T_ method (ΔΔC_T_ method) was used to analyze relative gene expression changes from qRT-PCR data (Livak and Schmittgen, 2001). Statistical significance and SEM for qRT-PCR data were calculated from ΔC_T_ values. To compare two groups (experimental to control) Mann–Whitney test was used, given assumption of non-Gaussian distribution. Statistical analyses were conducted using GraphPad Prism version 7.0 (GraphPad Software). All data are expressed with n representing the number of animals and with significance at *p* < 0.05.

## Results

### IUGR, hyperoxia, and IUGR/hyperoxia have distinct effects on the brain transcriptome

**Figure 1.**
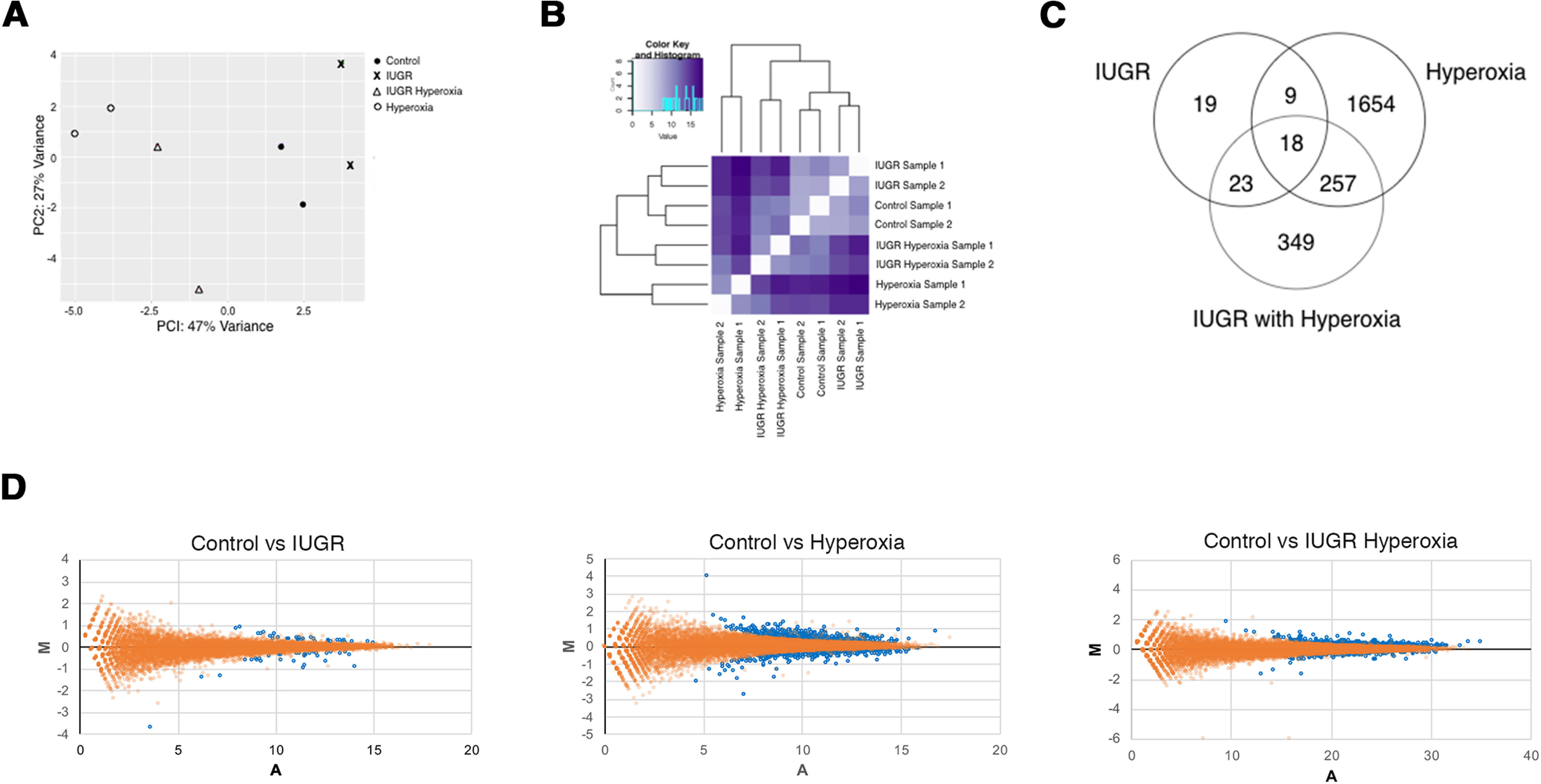
Comparison of RNA-seq gene expression data between control, IUGR, hyperoxia, and IUGR/hyperoxia. ***A***, PCA plot of gene expression showing variance between groups, *n* = 2 pooled samples. ***B***, Heatmap demonstrating difference in gene expression between groups/samples. Dark purple represents most variance and white least variance. ***C***, Venn diagram of significant DEGs in IUGR, hyperoxia, and IUGR/hyperoxia (FDR adj *p* < 0.05). ***D***, MA plots showing relationship between control versus IUGR, control versus hyperoxia, and control versus IUGR/hyperoxia. M (log ratio) and A (mean of normalized read counts). Orange dots represent genes that are not significantly different and blue spots represent genes that are significantly different (*p* < 0.05).

**Figure 2.**
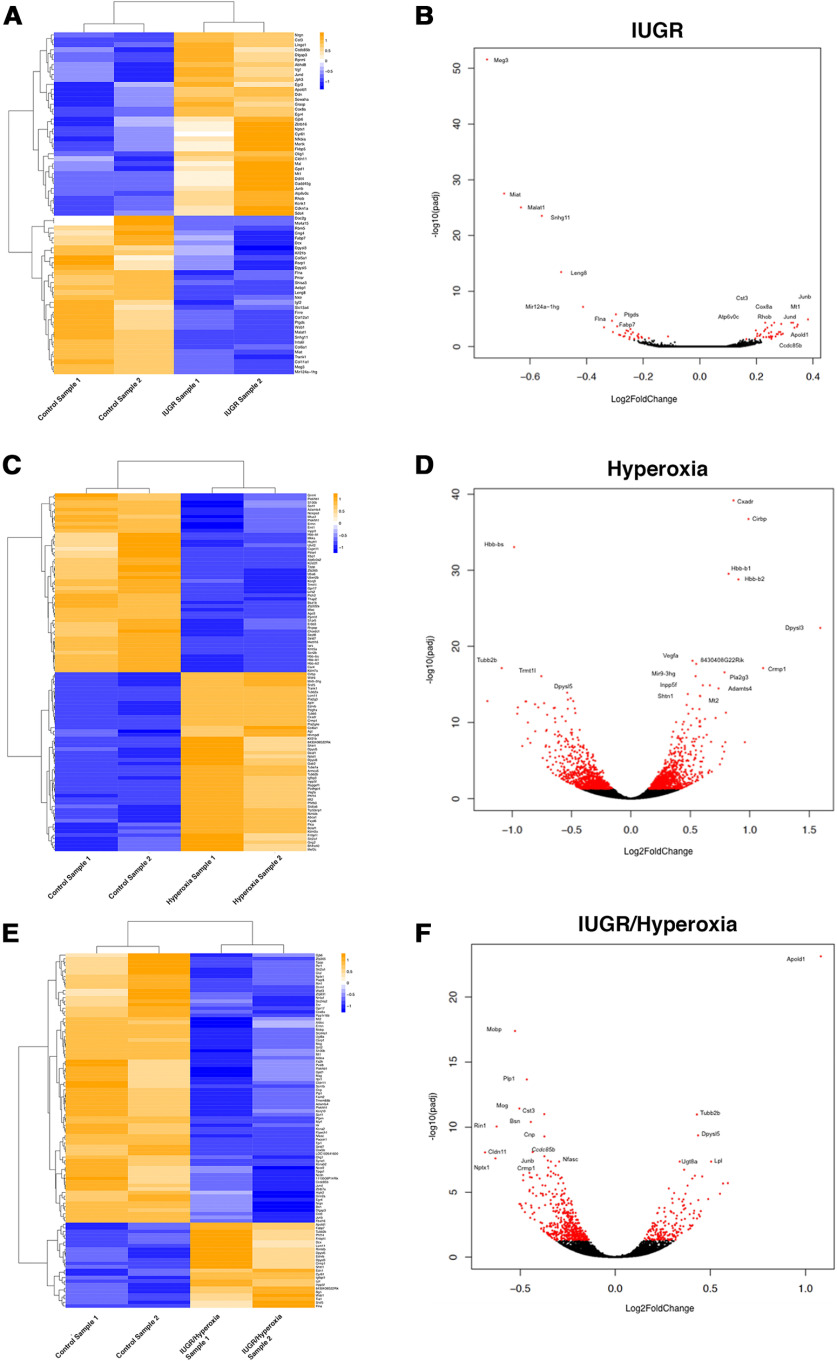
Comparison of significant DEGs in IUGR, hyperoxia, and IUGR/hyperoxia to control (FDR adj *p* < 0.05). ***A***, Heatmap of all significant DEGs between control and IUGR samples. Genes with similar expression patterns are clustered together (upregulated genes are dark orange and downregulated genes are dark blue). ***B***, Volcano plot of all DEGs in IUGR (significant DEGs with top 20 DEGs are red and labeled, non-significant DEGs in dataset are black). ***C***, Heatmap of top 100 significant DEGs between control and hyperoxia samples. ***D***, Volcano plot of all DEGs in hyperoxia group [top 25 significant (red) DEGs are red and labeled]. ***E***, Heatmap of top 100 significant DEGs between control and IUGR/hyperoxia samples. ***F***, Volcano plot of all DEGs in IUGR/hyperoxia group (top 25 significant DEGs are red and labeled).

To test whether IUGR and postnatal hyperoxia alters expression of genes associated with oligodendrogliogenesis or myelination, we performed RNA-seq in a pilot experiment using total RNA pooled from a small number of P14 brains. P14 was chosen as it is the midpoint of myelination in rodents and allowed for sufficient postnatal hyperoxia exposure. We performed differential expression analysis (DEA) and compared three experimental groups against control: IUGR, hyperoxia, and IUGR/hyperoxia (Fig. 1*A*,*B*). We observed distinct gene expression between groups (Fig. 1*A–C*). IUGR had the fewest DEGs compared with control (69; FDR adj *p* < 0.05), while hyperoxia had the greatest (1924). IUGR/hyperoxia had 647 DEGs (Fig. 1*C*,*D*). There was minimal overlap between groups, and only 18 DEGs common to all. Top 20 upregulated and downregulated DEGs also differed between groups, including directionality of expression, with prominent downregulation in IUGR/hyperoxia (Fig. 2).

### IUGR and hyperoxia have different effects on OL genes

**Figure 3.**
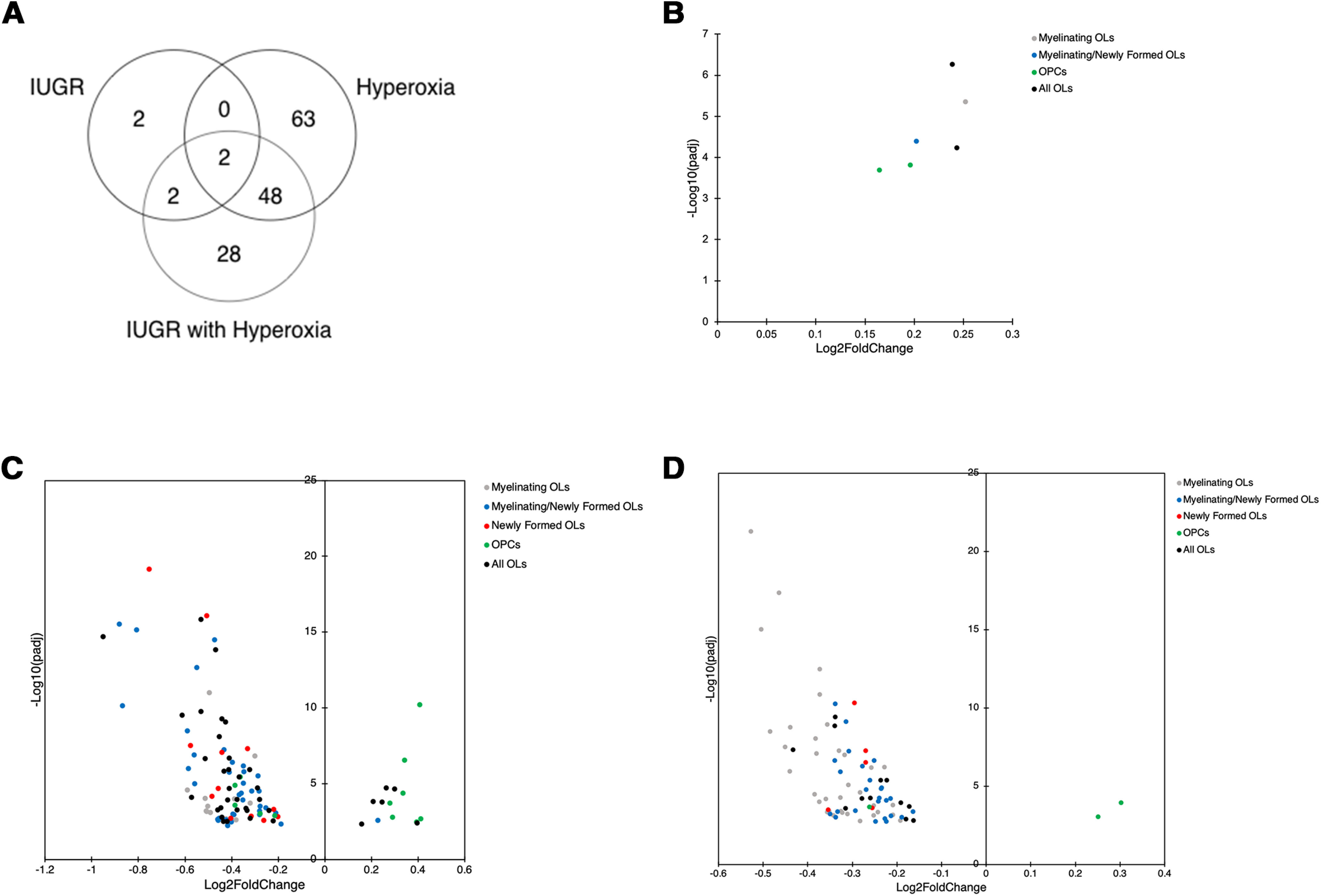
Comparison of differentially expressed OL genes (OLDEGs) in IUGR, hyperoxia, and IUGR/hyperoxia. ***A***, Venn diagram of significant OLDEGs in IUGR, hyperoxia, and IUGR/hyperoxia (FDR adj *p* < 0.05). ***B***, Scatter plot of significant OLDEGs in IUGR. Fold difference between log2 normalized expression in IUGR and control plotted versus -log10 adjusted *p* value. OLDEGs subcategorized by type of cell in OL lineage they are expressed by (gray: myelinating OL, blue: myelinating/newly formed OLs, red: newly formed OLs, green: OPCs, or black: all OLs). ***C***, Scatter plot of significant OLDEGs in hyperoxia. ***D***, Scatter plot of significant OLDEGs in IUGR/hyperoxia.

To determine whether expression changes lead to WMI in the three groups, we evaluated DEGs specifically expressed by oligodendroglia (OLDEGs) in the pooled data set. DEGs were categorized by brain cell type using a transcriptome database (Zhang et al., 2014). We found that IUGR, hyperoxia, and IUGR/hyperoxia demonstrated distinct patterns (Fig. 3*A*). IUGR yielded 6 OLDEGs, hyperoxia yielded 113, while IUGR/hyperoxia yielded 80, with minimal overlap between groups. Notably, 63 OLDEGs were exclusively altered in hyperoxia and 28 exclusively expressed in IUGR/hyperoxia. We next categorized OLDEGs by when in the lineage expression occurred, e.g., mature myelinating OLs, myelinating/newly formed OLs, newly formed OLs, OPCs, or expression seen at all stages of differentiation (Table 1). IUGR showed upregulation of 6 OLDEGs throughout the lineage (Fig. 3*B*). Hyperoxia demonstrated predominant downregulation of OLDEGs in myelinating/newly formed OLs (Fig. 3*C*). IUGR/hyperoxia demonstrated downregulated OLDEGs primarily in later maturation stages (Fig. 3*D*), 75% of genes expressed exclusively by mature myelinating and myelinating/newly formed OLs. DEGs with the largest magnitude Log2 fold change and lowest FDR adjusted *p* values were myelin specific: *MoBP*, *Plp1*, *Mog*, and *Cnp* (Table 2).

**Table 1:** DEGs categorized by OL cell type in IUGR, hyperoxia, and IUGR/hyperoxia compared with control

Experimental group	Type of OL cell	Number of genes (percentage)
IUGR	Mature myelinating OL	1/6 (16.67%)
	Myelinating/Newly formed OL	1/6 (16.67%)
	Newly formed OL	0 (0%)
	OPCs	2/6 (33.33%)
	All OL (non-specific expression)	2/6 (33.33%)
		
Hyperoxia	Mature myelinating OL	15/113 (13.27%)
	Myelinating/Newly formed OL	37/113 (32.74%)
	Newly formed OL	10/113 (8.85%)
	OPCs	14/113 (12.39%)
	All OL (non-specific expression)	37/113 (32.74%)
IUGR with hyperoxia	Mature myelinating OL	35/80 (43.75%)
	Myelinating/Newly formed OL	25/80 (31.25%)
	Newly formed OL	5/80 (6.25%)
	OPCs	3/80 (3.75%)
	All OL (non-specific expression)	12/80 (15%)

**Table 2:** Top 10 differently expressed myelin genes in IUGR/hyperoxia compared with control in RNASeq dataset

Gene symbol	Log2 fold change	FDR adj *p* value
*Mobp*	−0.526952566	5.26E-22
*Plp1*	−0.464800028	4.34E-18
*Mog*	−0.50406988	9.40E-16
*Cnp*	−0.372706581	3.38E-13
*Mag*	−0.355944387	1.13E-09
*Myrf*	−0.329926462	5.55E-08
*Mal*	−0.384694903	3.41E-05
*Mbp*	−0.326101762	0.000401
*Opalin*	−0.311350925	0.000604
*Omg*	−0.19212208	0.00159

### Hyperoxia with and without IUGR decreases myelin gene network expression

**Figure 4.**
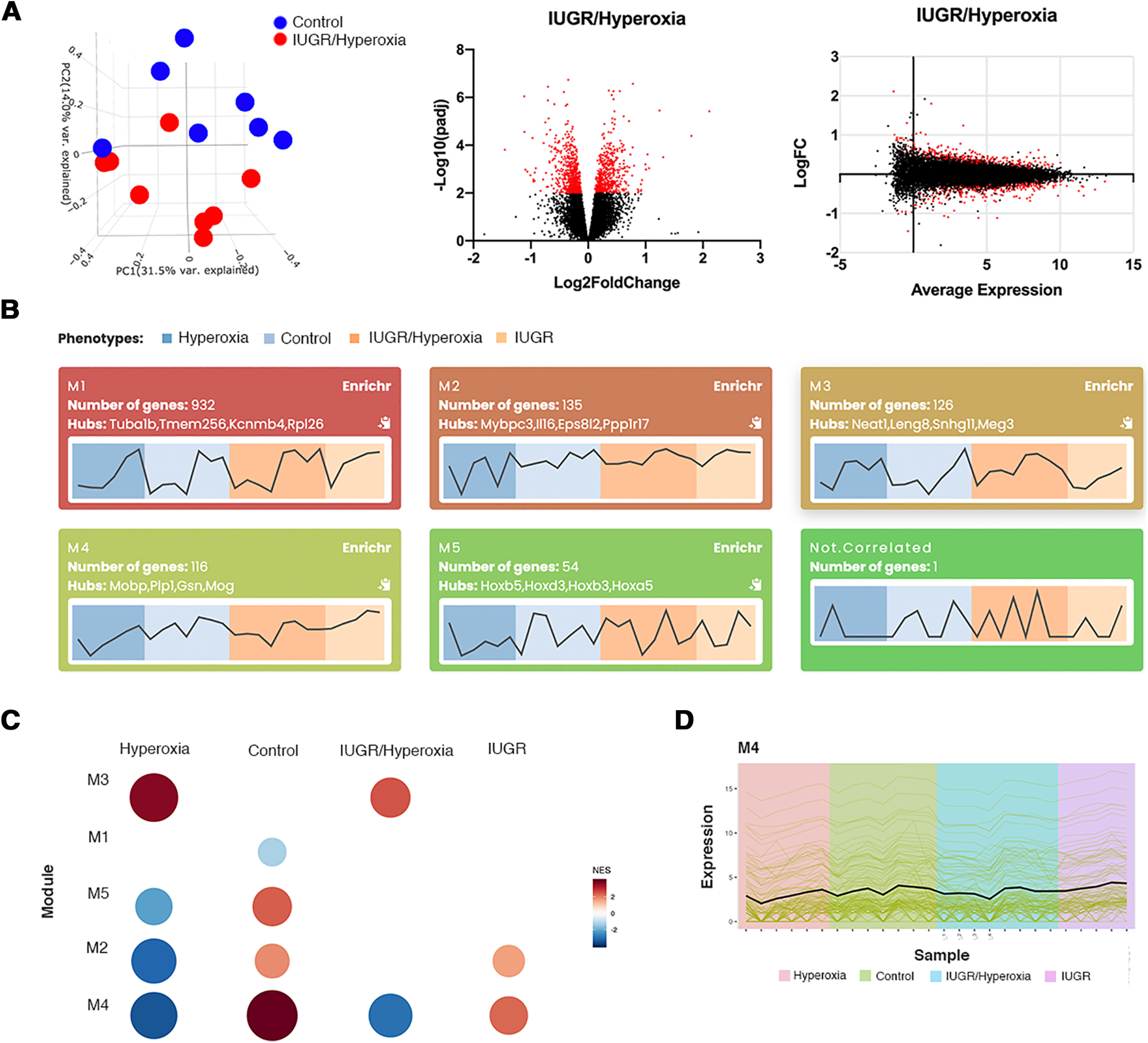
Differential gene analysis and co-expression analysis of expanded non-pooled data. ***A***, PCA plot comparing control versus IUGR/hyperoxia samples (control *n* = 7; IUGR/hyperoxia *n* = 8). Volcano plot of all DEGs in IUGR/hyperoxia group, significant DEGS (*p* < 0.05) are in red. MA plot showing relationship between control versus IUGR/hyperoxia. red represents genes that are significantly different (*p* < 0.05). ***B***, CemiTool Module profile plots for modules M1–M6. Number of genes and hub genes displayed for each module. Black line in each plot indicates median expression activity of genes in the module. Experimental groups are color coded. ***C***, Gene set enrichment analysis showing the M4 module activity on each class of samples. The size and color of the circle represents the normalized enrichment score (NES). ***D***, Profile plot for M4. The black line represents the median expression activity of all genes in the module. Samples are shown in the *x*-axis and colors represent the different experimental groups.

We repeated the RNA-seq using a larger sample size of individual (non-pooled) samples. Focusing on the IUGR/hyperoxia data, as our pooled samples had shown the greatest effect on WMI in this group, we again found distinct gene expression compared with control (Fig. 4*A*). While DEA identifies a large number of genes that differ between groups, it does not give information on interconnections between DEGs. To address this, we performed unsupervised gene co-expression analysis on the non-pooled RNA sequencing data with CEMiTool (Russo et al., 2018). CemiTool generated five modules highly correlated with the data set (Fig. 4*B*). Module 4 (M4) was significantly enriched with 134 genes that were identified to be related to myelination by the hub genes: *MoBP*, *Plp1*, *Gsn*, and *Mog* (Fig. 4*B*). Notably, activity in M4 was lower in both hyperoxia and IUGR/hyperoxia (Fig. 4*C*,*D*) as demonstrated by statistically significant adjusted *p* values and normalized enrichment scores (Table 3). These findings support the results from the DEA performed on our pooled samples and add further evidence that specific myelin genes are differentially expressed following these exposures (Chang et al., 2018).

**Table 3:** Co-expression modules (M1–M5) with adjusted *p* values and normalized enrichment scores (NES) for control, IUGR, hyperoxia, and IUGR/yyperoxia

Module	Control adj *p* value	Control NES	Hyperoxia adj *p* value	Hyperoxia NES	IUGR/hyperoxia adj *p* value	IUGR/hyperoxia NES	IUGR adj *p* value	IUGR NES
M1	0.00396	−1.27	0.25	1.34	0.74373	0.94	1	0.71
M2	0.00411	1.87	0.0002	−3.12	0.0643	1.32	0.00025	1.61
M3	0.15473	−1.21	0.00169	3.61	0.00053	2.47	0.625	−6.83
M4	0.00396	3.94	0.0002	−3.38	0.00413	−2.95	0.00025	2.27
M5	0.00396	2.38	0.0002	−2.13	0.0643	1.4	0.15152	−1.39

### Unique gene regulators identified in hyperoxia and IUGR/hyperoxia

**Figure 5.**
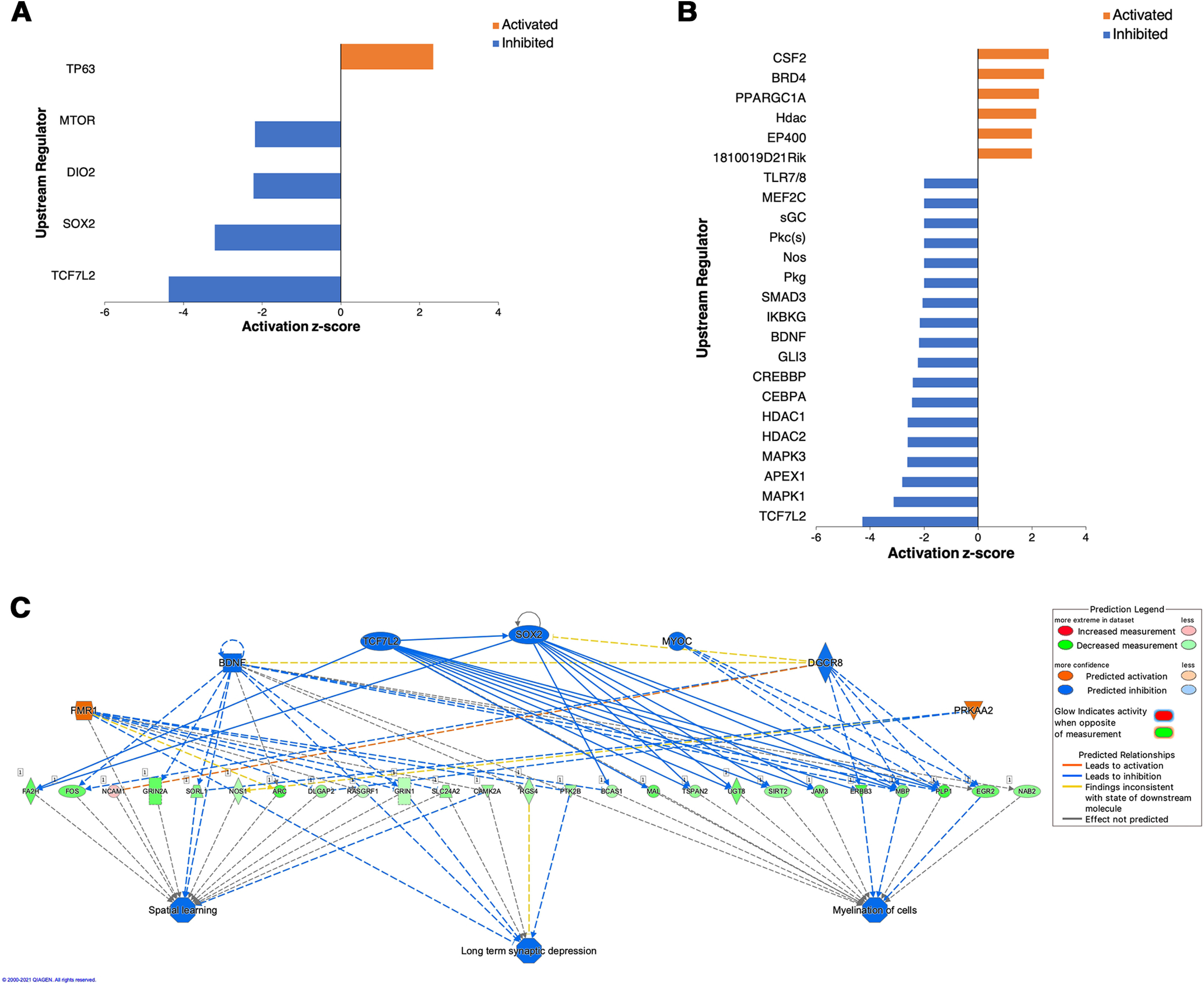
Upstream regulators identified with IPA from non-pooled RNA-seq data. ***A***, Upstream regulators identified in hyperoxia alone with *p* value of overlap < 0.05; orange: upstream regulators with *z* scores ≥ 2 (activated) and blue: upstream regulators with *z* scores ≤ −2 (inhibited). ***B***, Upstream regulators identified in IUGR/hyperoxia with *p* value of overlap < 0.05 and *z* scores ≥ 2 or ≤ −2. ***C***, Regulator Effects identified altered regulators and networks in IUGR/hyperoxia. Upstream regulators are located at the top of the network, target genes are in the middle of network, and predicated disease or function in the bottom of network.

We applied IPA to our non-pooled dataset to determine potential gene relationships and upstream regulators (Krämer et al., 2014). Using *p* < 0.05 and the additional cutoff of predicted *z* score ≥ 2 or ≤ −2, IPA identified no upstream regulators in IUGR, 5 upstream regulators in hyperoxia (Fig. 5*A*), and 25 in IUGR/hyperoxia (Fig. 5*B*). TCF7L2 was the only regulator in common between hyperoxia (*p* = 1.95E-12, *z* = −4.375) and IUGR/hyperoxia (*p* = 3.97E-8, *z* = −4.28). The Regulator Effects algorithm in IPA was next applied to the IUGR/hyperoxia data. This algorithm connects upstream regulators, dataset molecules and downstream functions/diseases, to generate hypotheses that can explain how the activation/inhibition of an upstream regulator affects downstream target molecule expression, and the impact of molecular expression on functions/diseases (Krämer et al., 2014). In addition to TCF7L2, predicted upstream regulators BDNF (*p* = 0.0002, *z* = −2.189), SOX2 (*p* = 5.64E-5, *z* = −1.672), MYOC (*p* = 0.0006, *z* = −1), DGCR8 (*p* = 5.93E-8, *z* = −1.369), and FMR1 (*p* = 2.9E-10, *z* = 3.578) were suggested to inhibit myelination (Fig. 5*C*).

### Perturbed myelin gene expression varies regionally in IUGR, hyperoxia, and IUGR/hyperoxia

**Figure 6.**
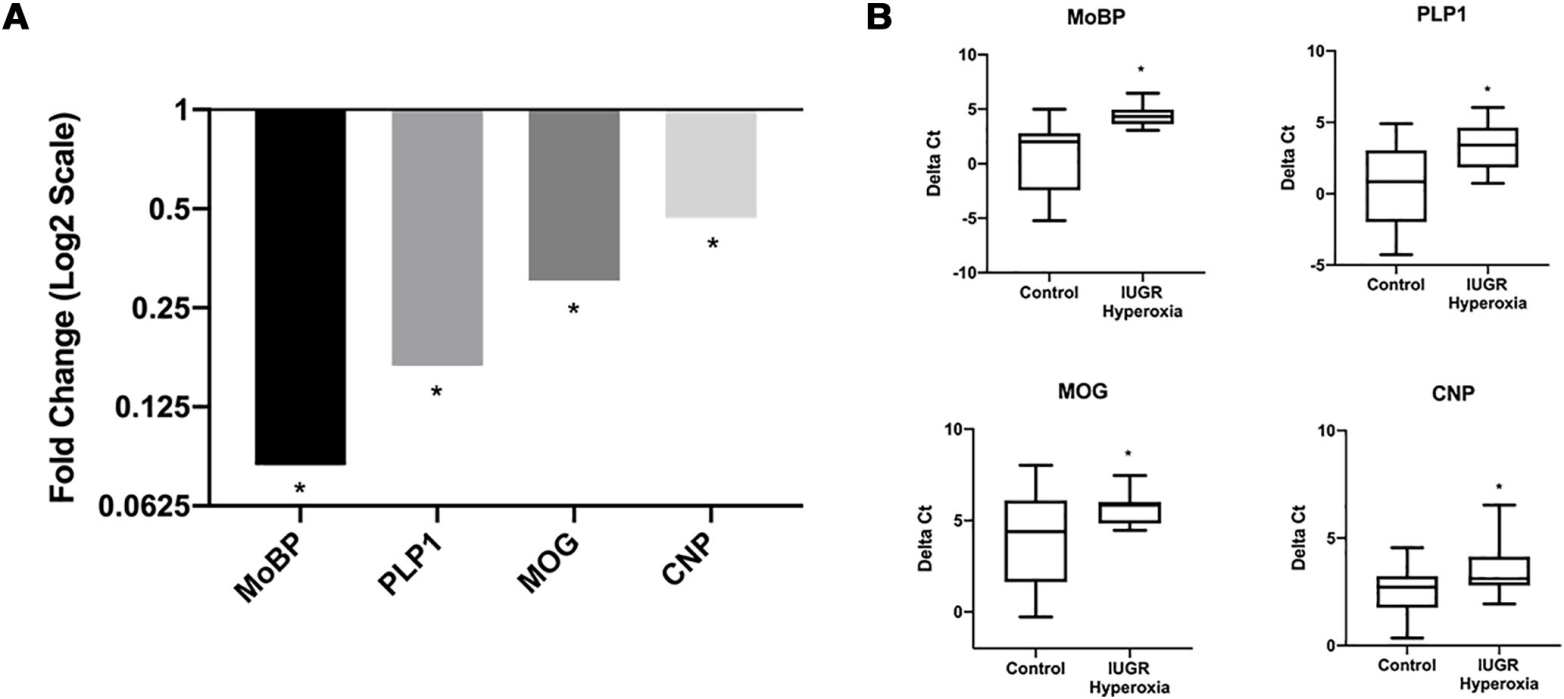
Validation of four most significantly downregulated myelin genes in IUGR/hyperoxia using qRT-PCR. ***A***, Relative fold change (*y*-axis is log2 scale) of *Mog*, *Plp1*, *MoBP*, and *Cnp* in IUGR/hyperoxia versus control calculated using ΔC_T_ method from qRT-PCR data (control *n* = 15 and IUGR/hyperoxia *n* = 14, significance *p <0.05 calculated from ΔC_T_ values). ***B***, Box plots showing δ Ct (ΔC_T_) of *Mog*, *Plp1*, *MoBP*, and *Cnp* in control and IUGR/hyperoxia which were used to calculate statistical significance; *Gapdh* used as the normalization gene (control *n* = 15 and IUGR/hyperoxia *n* = 14, significance *p <0.05).

**Figure 7.**
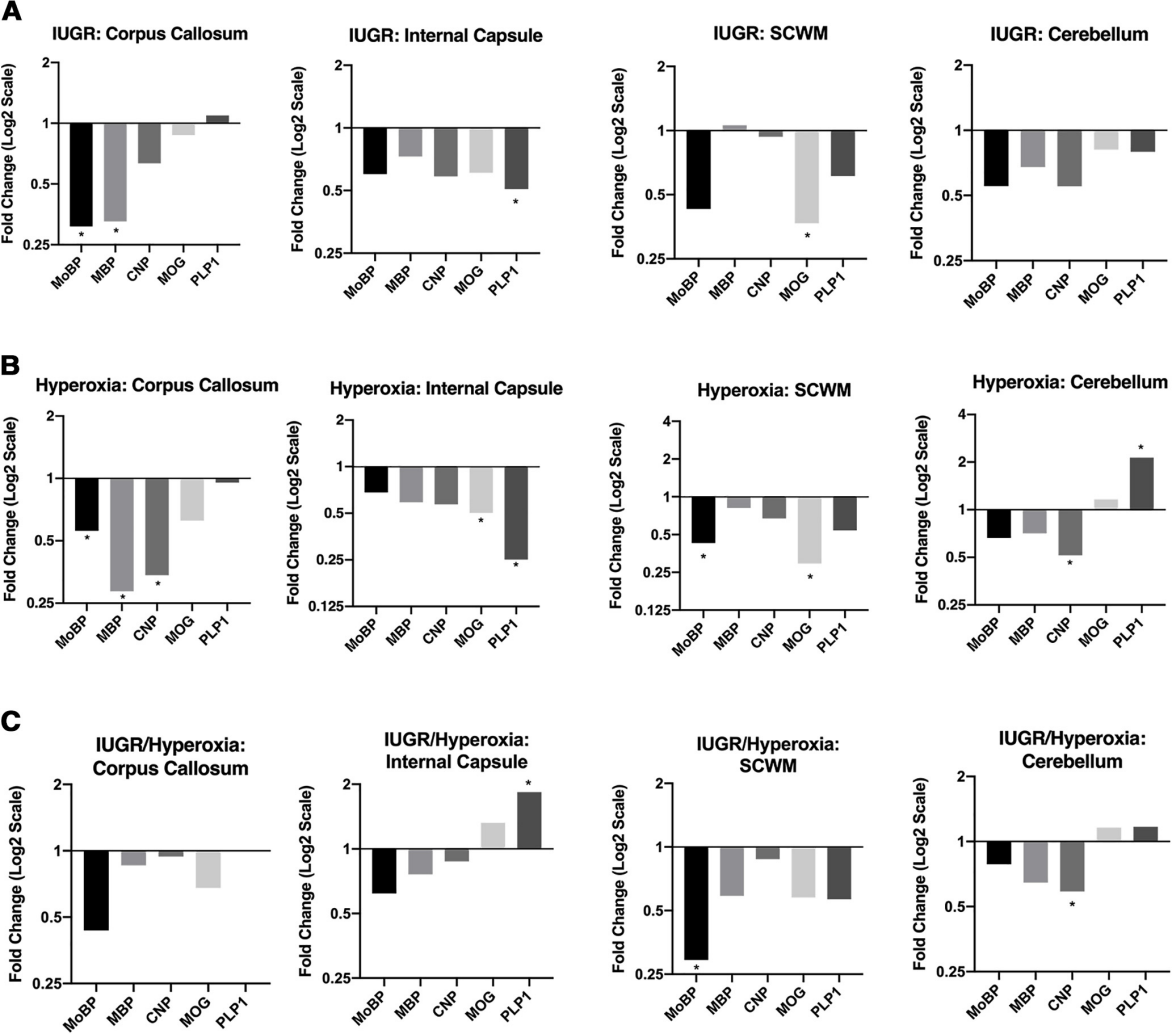
Myelin gene expression in different white matter regions of the brain (corpus callosum, internal capsule, SCWM, and cerebellum) measured with qRT-PCR at P14. ***A***, Relative fold change (log2 scale) of *MoBP*, *Mbp*, *Cnp*, *Mog*, and *Plp1* in IUGR versus control calculated using ΔC_T_ method. Corpus callosum (control *n* = 7; IUGR *n* = 9), internal capsule (control *n* = 6, IUGR *n* = 8), SCWM (control *n* = 5, IUGR *n* = 4), and cerebellum (control *n* = 4, IUGR *n* = 7), *p <0.05 calculated from ΔC_T_ values. ***B***, Relative fold change (log2 scale) of *MoBP*, *Mbp*, *Cnp*, *Mog*, and *Plp1* in hyperoxia versus control. Corpus callosum (control *n* = 7; hyperoxia *n* = 8), internal capsule (control *n* = 6, hyperoxia *n* = 8), SCWM (control *n* = 5, hyperoxia *n* = 7), and cerebellum (control *n* = 4, hyperoxia *n* = 7), *p <0.05. ***C***, Relative fold change (log2 scale) of *MoBP*, *Mbp*, *Cnp*, *Mog*, and *Plp1* in IUGR/hyperoxia versus Corpus callosum (control *n* = 7; IUGR/hyperoxia *n* = 6), internal capsule (control *n* = 6, IUGR/hyperoxia *n* = 6), SCWM (control *n* = 5, IUGR/hyperoxia *n* = 6), and cerebellum (control *n* = 4, IUGR/hyperoxia *n* = 6), *p <0.05.

To validate RNA-seq results, qRT-PCR was performed on four myelin genes that were found to be significantly downregulated in DEA: *MoBP*, *Plp1*, *Mog*, and *Cnp*. Using total RNA from control and IUGR/hyperoxia P14 hemispheres, we found all genes to be significantly downregulated, *p < 0.05 (Fig. 6). To determine whether specific white matter regions had greater myelin gene downregulation, we collected total RNA from four regions of interest at P14 for qRT-PCR: corpus callosum, internal capsule, SCWM, and cerebellum, then assessed expression of *MoBP*, *Plp1*, *Mog*, *Cnp*, and *Mbp*. Variable expression was seen in different regions for each treatment group. IUGR and hyperoxia showed statistically significant downregulation of OL genes in the corpus callosum and internal capsule that were not seen in entire hemisphere analysis (Fig. 7*A*,*B*). Surprisingly, IUGR/hyperoxia did not demonstrate significantly changed expression in white matter regions, although there was significant downregulation of *MoBP* in SCWM and *Cnp* in the cerebellum (Fig. 7*C*).

### Difference in myelin gene expression between sexes

**Figure 8.**
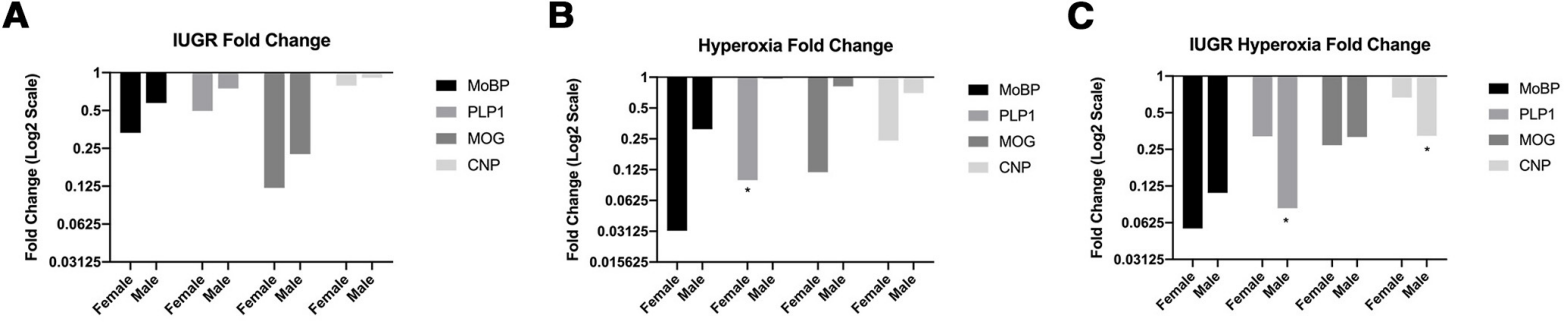
Sex comparison of myelin genes (*MoBP*, *Plp1*, *Mog*, *Cnp*) using qRT-PCR from RNA from P14 hemispheres. ***A***, Relative fold change (log2 scale) of IUGR (female *n* = 8, male *n* = 7) versus control (females *n* = 7, males *n* = 8) calculated using ΔC_T_ method, female versus male comparison. ***B***, Relative fold change of hyperoxia (female *n* = 7; male *n* = 5; *p < 0.05) versus control, female versus male comparison. ***C***, Relative fold change of IUGR/hyperoxia (female *n* = 7, male *n* = 7; *p < 0.05) versus control calculated using ΔC_T_ method, female versus male comparison.

To evaluate the impact sex has on WMI in IUGR and hyperoxia, qRT-PCR data for *MoBP*, *Plp1*, *Mog*, and *Cnp* from P14 hemispheres was separately analyzed by sex. There were no significant differences found between sexes in the control group. Nor were there significant differences between sexes found in IUGR, but there was a trend toward decreased myelin gene expression in females compared with males for all four genes (Fig. 8*A*). Hyperoxia showed decreased myelin gene expression in females compared with males that was statistically significant for *Plp1* (*p* = 0.01); the other three genes showed a trend toward decreased expression in females compared with males (Fig. 8*B*). In contrast, IUGR/hyperoxia showed statistically significant decreases in *Plp1* (*p* = 0.0006) and *Cnp* (*p* = 0.0076) in males compared with females (Fig. 8*C*).

## Discussion

WMI places IUGR infants at a higher risk of developing severe motor dysfunction including CP. Previous preclinical studies have shown that IUGR and postnatal oxygen exposure, individually and in combination, have adverse effects on the developing brain and result in WMI (Gerstner et al., 2008; Schmitz et al., 2011; Tolcos et al., 2011, 2017; Reid et al., 2012; Ramani et al., 2013; Ritter et al., 2013; Chang et al., 2018). Using a mouse model of placental insufficiency (Fung et al., 2011), we found distinct transcriptomic changes in the brain in three experimental groups: IUGR, hyperoxia, and IUGR/hyperoxia. Here, we observed that each group resulted in differences in gene transcription relating to white matter development and myelination. IUGR, hyperoxia, and IUGR/hyperoxia differed in the number and type of affected genes with minimal overlap in DEGs between each group. As all three study groups have previously shown altered myelination to some degree (Chang et al., 2018), it was somewhat surprising to find minimal overlap in differential gene expression. Additionally, the directionality of DEGs differed between groups, with prominent downregulation of genes in IUGR/hyperoxia that was not seen in IUGR nor hyperoxia alone. This finding suggests that disruption of white matter development likely occurs through distinct mechanisms and cellular interactions in each perturbation to the brain.

Distinct transcriptomic changes between groups were also seen in subanalysis of oligodendrocyte specific gene expression. Similar to what was seen when all cell types were examined, there was minimal overlap in OLDEGs between IUGR, hyperoxia, and IUGR/hyperoxia. The pattern of expression again showed predominant downregulation of OLDEGs in IUGR/hyperoxia. The predicted type of OL cell that was most affected also differed between groups. In hyperoxia, DEGs specific to newly formed/myelinating OLs were found to be the most affected. This is consistent with other studies showing transient loss of these cell types both in culture and in rat pups (Gerstner et al., 2006, 2008; Brill et al., 2017). In IUGR/hyperoxia, statistically significant DEGs were characteristic of cells later in the OL lineage, suggesting a specific effect on myelinating OLs with the combination of exposures. In fact, the DEGs with the lowest FDR adjusted *p* values and highest fold changes in the IUGR/hyperoxia dataset, before subanalysis, were myelin genes, including *MoBP*, *Plp1*, *Mog*, and *Cnp*. Unlike in IUGR/hyperoxia, the expression of these four myelin genes were not as significantly affected in IUGR or hyperoxia alone. This indicates that transcriptional changes in OLs are specific to the conditions of IUGR, hyperoxia, and IUGR/hyperoxia, lending insight into the differing type of WMI previously identified in these groups (Chang et al., 2018).

Of note, the majority of the significant DEGs in our DEA had fold changes <2 or >−2, a cutoff routinely used in RNA sequencing studies. One explanation, is that the brain contains a multitude of different cell types and the effect on oligodendrocytes, primarily perturbed in WMI, would be diluted by these other cells (∼80%) in the brain (Valerio-Gomes et al., 2018). As subtle changes in levels of RNAs can have biologically meaningful insights (Cantone et al., 2019), we chose to include all DEGs in our analysis if they satisfied the statistical cutoff of FDR adjusted *p* < 0.05. In doing so, we were able to discover significant downregulation of DEGs specific to oligodendrocytes. This supported our hypothesis that gene expression changes occur in IUGR and hyperoxia to reflect the WMI previously reported (Chang et al., 2018). Given these promising results, we performed DEA and co-expression analysis, and applied IPA using RNA from an expanded non-pooled data set. The subsequent analyses provided us with information on gene networks and potential mechanisms, adding to the understanding of transcriptome changes occurring in IUGR and hyperoxia.

Unsupervised gene co-expression analysis provides information on the interconnections between the DEGs that cannot be determined by DEA alone. It creates networks of genes (modules) by using the fact that genes participating in the same molecular and biological processes tend to show highly correlated expression patterns (co-expression; van Dam et al., 2018). Co-expression analysis has provided important biological insights into infectious (Janova et al., 2016), inflammatory (Jochems et al., 2018), and neurologic disease (Voineagu et al., 2011). Co-expression analysis has also been shown to enhance gene relationships that are only seen as modest gene changes in DEA (Abbassi-Daloii et al., 2020). Analysis of our non-pooled RNA-Seq dataset identified a network of 134 genes enriched with genes primarily involved in myelination. This network was decreased in hyperoxia and IUGR/hyperoxia compared with the control and IUGR groups. This supports our DEA results that genes related to myelination are specifically affected by hyperoxia and IUGR/hyperoxia.

While co-expression networks are able to identify correlations, indicating which genes are active simultaneously and likely biologically related, they do not provide information about causality or distinguish between regulatory/regulated genes (van Dam et al., 2018). Therefore, we next used IPA to identify potential upstream regulators and provide insight into the mechanism for WMI in the different exposure groups. In both hyperoxia and IUGR/hyperoxia, a pronounced downregulation of TCF7L2 signaling was seen. Downstream target molecules of TCF7L2 in our dataset included major myelin genes *MoBP*, *Cnp*, *Mog*, *Plp1*, and *Mbp*. TCF7L2 is a transcription factor specifically expressed in OLs during the time window that is critical for myelin formation, during the transition from OPCs to mature myelinating OLs (Fu et al., 2012; Lürbke et al., 2013; Hammond et al., 2015). It acts as a co-activator of β-catenin and is part of the canonical Wnt/β-catenin pathway, a well-known signaling pathway involved in neurogenesis and OL maturation (Gaesser and Fyffe-Maricich, 2016). Inhibition of TCF7L2 in hyperoxia and IUGR/hyperoxia can therefore explain the specific downregulation we found in newly formed and myelinating OLs. Dysregulation of the Wnt pathway has been implicated in other types of perinatal WMI including HI encephalopathy, periventricular leukomalacia, and prematurity (Fancy et al., 2009; Back, 2017), and thus it is unsurprising that it may be involved in WMI secondary to hyperoxia and IUGR/hyperoxia.

Our study also supports, as has been previously demonstrated, that hyperoxia in isolation results in WMI (Gerstner et al., 2008; Ramani et al., 2013; Ritter et al., 2013; Chang et al., 2018). TCF7L2, SOX2 and mTOR, which are known to be important in normal myelination, made up three out of the four upstream regulators identified in the hyperoxia group as inhibited. The transcription factor SRY-box 2 (SOX2) has been shown to be involved in OL proliferation and differentiation during postnatal brain myelination (Hoffmann et al., 2014). SOX2 also plays a role in CNS remyelination after injury and acts by recruiting adult OPCs (Zhao et al., 2015). The mTOR/Akt pathway is a signaling pathway known to be integral in many aspects of OL development including OPC differentiation, myelination, and survival (Narayanan et al., 2009; Gaesser and Fyffe-Maricich, 2016). Several studies report that levels of both myelin mRNAs and proteins are reduced following inactivation of mTOR signaling (Bercury et al., 2014; Lebrun-Julien et al., 2014; Wahl et al., 2014). Our findings now add specific transcription factors and pathways that may be involved in WMI because of postnatal hyperoxia exposure.

In contrast to IUGR and hyperoxia alone, a much larger number of upstream regulators were identified in IUGR/hyperoxia by IPA analysis. To understand the role of these additional upstream regulators in WMI, we used the regulator effects algorithm in IPA. This algorithm integrates results from the upstream regulator and downstream effects tools, to create hypotheses to explain how upstream regulators may cause a specific phenotype or outcome (Krämer et al., 2014). In addition to TCF7L2 and BDNF, which were identified in Upstream Regulator analysis, we were able to connect additional upstream effectors SOX2, MYOC, DGCR8, and FMR1, to the outcome of inhibited myelination. Similar to TCF7L2, the identification of BDNF involvement in IUGR/hyperoxia was unsurprising as this growth factor has been shown to pay a protective role in the neonatal brain following HI injury (Han and Holtzman, 2000; Chen et al., 2013). BDNF signals through oligodendrocyte-expressed TrkB that, in turn, activates the MAPK/Erk pathways to promote OL differentiation and myelination (Fletcher et al., 2018). *Bdnf* knock-out mice exhibit significant decreases in the expression of MBP and reduced mRNA transcripts of MBP and PLP in the hippocampus and cortex (Vondran et al., 2010; Gonsalvez et al., 2016; Fletcher et al., 2018). Additionally, BDNF heterozygous null mice demonstrate significant reductions in expression of MBP, PLP, MAG, and MOG in forebrain, corpus callosum, spinal cord and optic nerves (Djalali et al., 2005). The MAPK/Erk signaling is implicated as a late-stage regulator of CNS myelination (Gonsalvez et al., 2016; Ishii et al., 2019) and has also been shown to be important for myelin maintenance throughout adulthood (Ishii et al., 2012). Inhibition of BDNF in IUGR/hyperoxia therefore correlates with the specific downregulation of DEGs we found in myelinating/newly formed and mature myelinating OLs.

An additional interesting gene identified was *Dgcr8* which regulates primary miRNA (miR) processing and has been found to be important in regulating progression of differentiation of oligodendroglia, myelin formation, and myelin maintenance (Lin et al., 2015). Alterations in miRs have been observed to change in several types of preterm brain injury. Differential expression of miRs was shown first in plasma of preterm infants with intraventricular hemorrhage (Chapman et al., 2018). Subsequently, changes in specific miRs within exosomes in CSF following posthemorrhagic hydrocephalus have been shown (Spaull et al., 2019). Proinflammatory miRs have also been found to correlate with oxidized hemoglobin metabolites and heme in CSF after intraventricular hemorrhage (Fejes et al., 2020). Additionally, miRs have been widely studied in HI encephalopathy in term neonates, in serum, dried blood spots and whole blood, as potential biomarkers of injury severity (Ponnusamy et al., 2016; Wang et al., 2018; Zhang et al., 2020). Specific to WMI, it was shown that mature miRs suppress the regenerative OL response to perinatal HI (Birch et al., 2014). Thus, differential expression of genes involved in miR processing is likely to have effects on white matter development.

These additional regulators identified in IUGR/hyperoxia by the regulator effector algorithm had *z* scores that were not ≥ 2 or ≤−2 so may otherwise have gone overlooked. Many of these upstream regulators converge to inhibit MBP and PLP1, which explains the pronounced downregulation of these genes in both DEA and co-expression analysis. The unique dysregulation identified by IPA at multiple points, from transcription, processing of miR, and OL cellular signaling pathways in IUGR/hyperoxia, supports our idea of a multihit hypothesis of WMI with this combined insult. It also seems likely that each of these steps, that are known to be independently critical for myelination, are likely intertwined, and that appropriate and effective myelination occurs only when normal interactions are maintained.

We expected to see a more pronounced downregulation of myelin genes in IUGR/hyperoxia within isolated large white matter tracts, however, this was not observed. Instead, the most significant downregulated myelin genes, including *MoBP*, *Plp1*, *Cnp*, *Mog*, and *Mbp*, that we observed in entire hemisphere showed less significant changes in the corpus callosum, internal capsule, SCWM, and cerebellum. One potential explanation is that IUGR/hyperoxia affects the descending motor tracts, including the corticospinal tract, that run through the internal capsule later in development. A significant decrease in WM fiber length and volume in the internal capsule has been demonstrated on MRI with DTI at P28 in IUGR mice exposed to postnatal hyperoxia (Chang et al., 2018). Further investigation into the effect that timing of injury has on gene dysregulation would help us better understand the injury that occurs in this model. Specifically, postnatal hyperoxia exposure may affect gene expression differently, the longer the exposure time period. In addition, investigating different regions of the brain at earlier and later time points would also better define the injury that occurs in this model, as myelination of the developing brain occurs in the rostral to caudal direction. Thus, examination of a single time point is a limitation of this study.

Clinical and animal studies have shown that sex may play a role in neonatal neurodevelopmental outcomes. A number of clinical studies have identified male sex as an independent risk factor for poor neurodevelopmental outcomes in prematurity (Hintz et al., 2006; Sunny et al., 2020) and HI injury (Mirza et al., 2015; Narang et al., 2019). A sex bias has also been identified in children with CP, with males being more affected than females (Johnston and Hagberg, 2007). Additionally, potential sex differences have been shown in IUGR and following neonatal oxygen exposure. In IUGR, clinical and animal studies have shown contrasting results, some demonstrating male sex as a risk factor (Parkinson et al., 1981; Fung et al., 2012) and others finding no difference or female sex as a risk factor (Reid et al., 2012; Streimish et al., 2012) for poor neurodevelopmental outcomes. In our study, we found no statistical difference between sexes in myelin gene expression in IUGR. Evaluation of sex-differences following hyperoxia exposure in the literature is more limited. In one study, the cellular functions related to energy metabolism, stress, response, and maturation because of oxidative stress were shown to be more pronounced in male versus female-derived OPCs that were exposed to high oxygen for 24 h (Sunny et al., 2020). In contrast, we demonstrate downregulation of myelin genes in females compared with males following hyperoxia exposure. One potential explanation is that, in our study, our animals were exposed to oxygen over a longer time period of 14 d. The combination of IUGR with hyperoxia resulted in significant downregulation of myelin genes in males compared with females. This is consistent with the male sex being a risk factor for worse neurodevelopment outcomes in other types of perinatal brain injury.

Overall, these findings highlight the complex nature of perinatal brain injury. They also underscore the detrimental effect that oxygen exposure can have on the developing white matter of IUGR infants. Hyperoxia is well known to be implicated in the pathogenesis of bronchopulmonary dysplasia and retinopathy of prematurity (Saugstad, 2001; Weinberger et al., 2002). Our study now adds to the increasing evidence that hyperoxia negatively influences brain maturation and development and results in WMI (Felderhoff-Mueser et al., 2004, 2005; Gerstner et al., 2008; Sunny et al., 2020). Additionally, our study importantly, indicates specific gene networks that contribute to previously demonstrated findings of abnormal myelin formation and motor dysfunction in IUGR and hyperoxia (Chang et al., 2018). The identification of differential gene expression leading to multiple dysregulated signaling pathways known to be integral to CNS myelination, including the Wnt/β-catenin and MAPK/Erk pathways in IUGR/hyperoxia, lend insight into how multiple perinatal exposures result in WMI. These findings further stress the need to temper use of therapeutic oxygen in growth restricted infants to minimize WMI.

## Synthesis

Reviewing Editor: Christophe Bernard, INSERM & Institut de Neurosciences des Systèmes Decisions are customarily a result of the Reviewing Editor and the peer reviewers coming together and discussing their recommendations until a consensus is reached. When revisions are invited, a fact-based synthesis statement explaining their decision and outlining what is needed to prepare a revision will be listed below. The following reviewer(s) agreed to reveal their identity: Eric Benner.

Reviewer #1

The manuscript is an original study on the combined effects of IUGR and hyperoxia treatment on myelination. The authors used a previously described model of IUGR and hyperoxia and performed a transcriptomic analysis on the genes that are differentially expressed in each of these exposures along with their combination. Their hypothesis that hyperoxia treatment in the NICU is responsible to a degree for white matter injury is valid, and with the ability to perform large-scale genetic analyses, it feels like a worthy endeavor. The genetic analysis itself is very thorough. There is a detailed explanation of the pathways affected, in the discussion. Overall, the authors take the IUGR/ hyperoxia model described by Chang et al one step further by proving that different genes are expressed in each harmful exposure (IUGR, hyperoxia and their combination) and result in different types of white matter injuryu, as documented by Chang et al.

Summary of Strengths and Weaknesses

The manuscript is an original study on the combined effects of IUGR and hyperoxia treatment on myelination. The authors used a previously described model of IUGR and hyperoxia and performed a transcriptomic analysis on the genes that are differentially expressed in each of these exposures along with their combination. Their hypothesis that hyperoxia treatment in the NICU is responsible to a degree for white matter injury is valid, and with the ability to perform large-scale genetic analyses, it feels like a worthy endeavor. The genetic analysis itself is very thorough. Appreciate the detailed explanation of the pathways affected, in the discussion. Overall, the authors take the IUGR/ hyperoxia model described by Chang et al, one step further, by proving that different genes are expressed in each harmful exposure (IUGR, hyperoxia and their combination) and result in different types of WMI, as documented by Chang et al. However, the manuscript lacks punctuation in many sections, which make it difficult to understand. The phrasing could also be improved in places.

Main concerns:

Assignment of groups: The authors state that the IUGR group consisted of pups weighing less than the 10th percentile for sham weight, and these pups were defined as SGA. Does this mean that only 1/3 of the pups born to mothers receiving the analog, were included in the IUGR group? Furthermore, what was the percentage of vehicle pups that were born with weights greater than the 10th percentile? Were there pups born from dams receiving just ethanol, weighing less than the 10th percentile, that were not included in the study? If so, what is the reasoning for this exclusion? There is an issue with sample numbers- it is unclear how pups were assigned and also the overall sample size is small and needs to be increased given the small effects.

Pooled samples: The authors mentioned that they pooled RNA from different pups to create 2 samples per group. What was the reasoning for this? The sample size is small, as the authors acknowledge in the discussion, so why did the authors choose pooled RNA versus RNA from each pup, to have a bigger sample size? Could you please elaborate more on “decreasing biological variance”?

DEGs: The differentially expressed genes in the three groups had little overlap but it would be interesting to see a comparison of the few overlapping genes in IUGR, hyperoxia and IUGR with hyperoxia, instead of each of those groups with the control group. Perhaps a figure similar to figure 2?

2. Suggestions for Minor Revisions

Throughout the manuscript there is an obvious lack of punctuation marks, particularly commas. I would suggest that the authors thoroughly review the text, adding commas wherever appropriate to aid the clarity and comprehension of the manuscript.

Introduction

Paragraph 2 line 11: Consider removing “in these conditions” at the end of the sentence.

Paragraph 2 line 12: This sentence could be better rephrased as “The implicated alterations in gene expression and signaling pathways in the brain, that lead to abnormal white matter development, are now being investigated", since the term “modifications” implies deliberate change of the pathways or gene expression.

Paragraph 3 first sentence: Please provide a reference for the statement that not all IUGR infants develop CP.

Paragraph 3, line 7: “damage to developing white matter” should read “damage to the developing white matter”.

Paragraph 3 line 9: Don’t use the phrasing “long thought”. The fact that perinatal brain injury is the result of multiple cascades and exposures has been reported in many studies.

Paragraph 4 lines 8-9: Consider rewriting this sentence as follows “The combination of IUGR and hyperoxia also lead to more pronounced gait changes and white matter injury on MRI in adult mice”.

Paragraph 4 following sentence: Consider replacing “with these perinatal exposures” with “with each type of exposure”. Also remove “with the different exposures” at the end of the sentence.

Paragraph 5 lines 3-4: No need to use “alone” three times. This sentence should read: “We showed that IUGR and hyperoxia alone, resulted in distinct changes in gene expression, with hyperoxia affecting many pathways...” The authors have already made clear that they refer to the combination of IUGR and hyperoxia as ‘the combination of..” or “IUGR with hyperoxia”.

Methods

- Murine IUGR model

Paragraph 1 last sentence: This could be rephrased as: “This model does not require invasive surgery and it is physiologically relevant to human IUGR pregnancies”.

Paragraph 2, lines 7-8: The sentence “After pups were delivered spontaneously” could be rewritten as: “Following spontaneous delivery, pups were weighted...”

Paragraph 2, line 9: The sentence “Pups born to dams...” could be rewritten as: “...weighing less than 1.266g, which was below the 10th percentile for sham pup weight”.

Paragraph 2: Please provide the full term for “SGA” since it hasn’t been clarified previously in the manuscript.

Discussion

Paragraph 1, first sentence: The authors should rephrase “oxygen exposure in isolation”. This sentence would sound better if it read: “IUGR, high oxygen exposure and their combination” or something along these lines.

Paragraph 1, following sentence: Please remove “this” before WMI at the beginning of the sentence.

Paragraph 1, line 4: Please expand the term “placental insufficiency” by including the cause: excess thromboxane A2. It would be useful to provide information on the specific model used, in the beginning of the discussion.

Paragraph 5 third line before the end: “Dysregulation of the Wnt pathway been..” should read “Dyregulation of the Wnt pathway has been...”

Paragraph 7 line 15: “On-gong” should read “ongoing”.

Paragraph 7, line 16: Please replace “MS” with “multiple sclerosis”.

Paragraph 9 line 4: Please remove “of” in “showed less significant of changes”.

Paragraph 9 line 10: This sentence is quite long so please consider removing “including the corticospinal tract”.

Paragraph 9 last line: There are too many “and” in this sentence. Consider rewriting as follows: “Samples were composed of pooled tissue from several animals, which minimized biological variance. Our approach was further validated by qRT-PCR that was highly correlated with RNA-Seq findings”.

Paragraph 10: Please remove the part about “suggesting new potential therapeutic targets” since this paragraph acts as a conclusion, and although there is a lot of detail on the pathways involved there is no mention of using these pathways for therapeutics anywhere else in the manuscript.

Reviewer #2

Neonatal WMI is an important problem in the NICU. There are numerous risk factors known - including IUGR and hyperoxia. Here the authors combine these 2 model systems and perform RNA-seq to identify gene/pathway changes that may contribute. To my knowledge, this is the first study that I have seen using this approach in this clinically relevant model system.

The authors explore changes in gene regulation between IUGR and IUGR + hyperoxia to support a multi-hit hypothesis that infants exposed to multiple insults may be at higher risk for NDI, particularly in motor outcomes. The authors combined 2 previously characterized models - thromboxane analogue induced IUGR as well as hyperoxia exposure during periods of white matter development. The authors then apply RNA-seq techniques to identify changes in genes and larger genetic pathways in these individual models or when they are combined.

WMI is a significant problem in the NICU - it’s the most common brain injury leading to poor neurologic outcomes in this population. Moreover, IUGR is an important risk factor for the development of WMI and as stated by the authors not all IUGR infants develop this injury. For that reason, the attempt to identify additional factors that influence risk in IUGR infants is both important and laudable. The results presented here are intriguing and evocative but the n’s are too small and they don’t have an even distribution of sex - a known determinant in neurologic outcomes (in humans and animal models). To my knowledge, this work is a new analysis for IUGR + hyperoxia.

Summary of key findings:

• RNA-seq analysis found small changes in tissue gene expression in all 3 experimental groups. Interestingly, the greatest change in downregulation of genes controlling WM development and myelination occurred in the IUGR/Hyperoxia group.

• This study attempts to identify genes/pathways common to all 3 models and identify unique changes to the IUGR/Hyperoxia groups. The data presented support the existence of unique genetic changes within the IUGR and hyperoxia group.

• Applied IPA highlights genetic pathways that appear to be dysregulated following IUGR and hyperoxia exposures. Many of these pathways have been previously identified as regulators of myelination/maturation.

• RNA isolation and QC is standard.

• Analysis used a standard pipeline

• Validation assays used are standard

Major concerns:

1. The changes in gene expression from whole hemisphere analysis is very small. The authors address this in the discussion and I agree that the impact on oligodendrocyte gene expression can be diluted with this type of analysis. Given the small Log2 Fold changes observed (<0.5), it is imperative that the sample number be increased.

2. When compared to whole hemisphere changes, dissection of WM should exaggerate these changes in myelination gene expression due to the increased representation of the oligodendrocyte lineage in the tissue sample. As the authors note in the discussion, this was not the case. The authors suggest that other white matter regions (cortical or subcortical) may be more affected. I think it is important to make this determination. As is, this makes us less confident that the small changes observed in the RNA-seq assay are meaningful.

3. Effects of sex. Generally, the impact of sex on brain injury outcomes have been well-documented in both human infants and model systems. Is there sex variation by the exposure here? The same number of females/males in each of the groups should be used at a minimum. Ideally, males and females could be pooled and analyzed separately. Pooling of numbers isn’t consistent. How did they determine how to pool the animals in terms of sex?

4. Figure 4D (qPCR X RNA-seq r^2 = 0.75) is barely acceptable.

5. U-46619 does not appear to cross the placenta - based on 11-dehydrothromboxane B2 levels. Can they provide the reference to this data or provide the data itself. This is important because the authors used U-46619 exposed animals that were >10%ile as controls.

6. The observed changes in gene expression within tissues could be related to a loss of OL lineages or a lack of maturation. This is a big question within the field of neonatal WMI - does the author have any evidence as to which is contributing to the observations presented here?

Minor concerns:

1. Labeling in figures would make this easier to read. For example, in Figure 2, labeling heat maps or volcano plots with IUGR, hyperoxia, and hyperoxia + IUGR.

Bottom line, intriguing and evocative but the n’s are too small + they don’t have an even distribution of sex which could be a confounder - needs more work. If this were to be published as is, a thorough discussion on sex factors impacting neurologic outcomes in the discussion would be important.

Possible recommendation for future work is to attempt to flow sort oligodendrocyte lineage using commercially available Olig2-EGFP alleles to isolate cells of interest prior to RNA-seq analysis. This may assist in identifying changes that are diluted out in the current study.

## Author Response

Manuscript Instructions

- The species studied is not mentioned in the abstract. Please make sure to update both the

abstract in the article file and on the submission form.

Specified in the abstract and the submission form that a mouse model was used in these

experiments.

---------------------------------------------

Synthesis of Reviews:

Reviewer #1

The manuscript is an original study on the combined effects of IUGR and hyperoxia treatment

on myelination. The authors used a previously described model of IUGR and hyperoxia and

performed a transcriptomic analysis on the genes that are differentially expressed in each of

these exposures along with their combination. Their hypothesis that hyperoxia treatment in the

NICU is responsible to a degree for white matter injury is valid, and with the ability to perform

large-scale genetic analyses, it feels like a worthy endeavor. The genetic analysis itself is very

thorough. There is a detailed explanation of the pathways affected, in the discussion. Overall,

the authors take the IUGR/ hyperoxia model described by Chang et al one step further by

proving that different genes are expressed in each harmful exposure (IUGR, hyperoxia and their

combination) and result in different types of white matter injury, as documented by Chang et al.

Summary of Strengths and Weaknesses

The manuscript is an original study on the combined effects of IUGR and hyperoxia treatment

on myelination. The authors used a previously described model of IUGR and hyperoxia and

performed a transcriptomic analysis on the genes that are differentially expressed in each of

these exposures along with their combination. Their hypothesis that hyperoxia treatment in the

NICU is responsible to a degree for white matter injury is valid, and with the ability to perform

large-scale genetic analyses, it feels like a worthy endeavor. The genetic analysis itself is very

thorough. Appreciate the detailed explanation of the pathways affected, in the discussion.

Overall, the authors take the IUGR/ hyperoxia model described by Chang et al, one step further,

by proving that different genes are expressed in each harmful exposure (IUGR, hyperoxia and

their combination) and result in different types of WMI, as documented by Chang et al.

However, the manuscript lacks punctuation in many sections, which make it difficult to

understand. The phrasing could also be improved in places.

Main concerns:

Assignment of groups:

• The authors state that the IUGR group consisted of pups weighing less than the 10th

percentile for sham weight, and these pups were defined as SGA. Does this mean that

only 1/3 of the pups born to mothers receiving the analog, were included in the IUGR

group?

Yes, only 1/3 of the pups born to dam’s receiving the analog were included in the IUGR

group.

• Furthermore, what was the percentage of vehicle pups that were born with weights

greater than the 10th percentile?

For an average litter size of 6, approximately 3/4 of vehicle pups in each litter were > 10th

percentile in weight. 2

• Were there pups born from dams receiving just ethanol, weighing less than the 10th

percentile, that were not included in the study? If so, what is the reasoning for this

exclusion?

Pups that were born that were naturally SGA were not included as we could not say that

these pups were small secondary to placental insufficiency.

• There is an issue with sample numbers- it is unclear how pups were assigned and also

the overall sample size is small and needs to be increased given the small effects.

Dams were assigned to vehicle or thromboxane, thus offspring were assigned to the

same treatment groups. Pups from vehicle groups that had birthweights <10th%ile were

not analyzed. Pups from thromboxane groups that had birthweights >10th%ile were not

analyzed. A second RNA sequencing experiment was performed for this revision with an

expanded sample size of n = 5-8 for each experimental group.

Pooled samples:

• The authors mentioned that they pooled RNA from different pups to create 2 samples

per group. What was the reasoning for this? The sample size is small, as the authors

acknowledge in the discussion, so why did the authors choose pooled RNA versus RNA

from each pup, to have a bigger sample size? Could you please elaborate more on

"decreasing biological variance”?

Pooled samples RNA samples were used in our initial experiment to increase efficiency,

decrease biological variance, and given the support of this approach in the literature

(Assefa, et al, 2020). For this revision, we performed a second RNA sequencing

experiment with an expanded sample size of individual (non-pooled) samples.

Additionally, the qRT-PCR validation experiments were also performed with non-pooled

samples.

• DEGs: The differentially expressed genes in the three groups had little overlap but it

would be interesting to see a comparison of the few overlapping genes in IUGR,

hyperoxia and IUGR with hyperoxia, instead of each of those groups with the control

group. Perhaps a figure similar to figure 2?

It definitely would be interesting to compare the three groups. However, since there were

only 18 differentially regulated genes (DEGs) in common between the 3 groups we

chose not to include a separate figure in the manuscript.

2. Suggestions for Minor Revisions

Throughout the manuscript there is an obvious lack of punctuation marks, particularly commas. I

would suggest that the authors thoroughly review the text, adding commas wherever

appropriate to aid the clarity and comprehension of the manuscript.

The grammatical revisions suggested below by the reviewer were made as possible in the

updated manuscript text.

Introduction

Paragraph 2 line 11: Consider removing “in these conditions” at the end of the sentence.

Paragraph 2 line 12: This sentence could be better rephrased as “The implicated alterations in

gene expression and signaling pathways in the brain, that lead to abnormal white matter

development, are now being investigated", since the term “modifications” implies deliberate

change of the pathways or gene expression.3

Paragraph 3 first sentence: Please provide a reference for the statement that not all IUGR

infants develop CP.

References were added to the manuscript.

Paragraph 3, line 7: “damage to developing white matter” should read “damage to the

developing white matter”.

Paragraph 3 line 9: Don’t use the phrasing “long thought”. The fact that perinatal brain injury is

the result of multiple cascades and exposures has been reported in many studies.

Paragraph 4 lines 8-9: Consider rewriting this sentence as follows “The combination of IUGR

and hyperoxia also lead to more pronounced gait changes and white matter injury on MRI in

adult mice”.

Paragraph 4 following sentence: Consider replacing “with these perinatal exposures” with “with

each type of exposure”. Also remove “with the different exposures” at the end of the sentence.

Paragraph 5 lines 3-4: No need to use “alone” three times. This sentence should read: “We

showed that IUGR and hyperoxia alone, resulted in distinct changes in gene expression, with

hyperoxia affecting many pathways...” The authors have already made clear that they refer to

the combination of IUGR and hyperoxia as ’the combination of..” or “IUGR with hyperoxia”..

Methods

- Murine IUGR model

Paragraph 1 last sentence: This could be rephrased as: “This model does not require invasive

surgery and it is physiologically relevant to human IUGR pregnancies”.

Paragraph 2, lines 7-8: The sentence “After pups were delivered spontaneously” could be

rewritten as: “Following spontaneous delivery, pups were weighted...”

Paragraph 2, line 9: The sentence “Pups born to dams...” could be rewritten as: “...weighing less

than 1.266g, which was below the 10th percentile for sham pup weight”.

Paragraph 2: Please provide the full term for “SGA” since it hasn’t been clarified previously in

the manuscript.

Discussion

Paragraph 1, first sentence: The authors should rephrase “oxygen exposure in isolation”. This

sentence would sound better if it read: “IUGR, high oxygen exposure and their combination” or

something along these lines.

Paragraph 1, following sentence: Please remove “this” before WMI at the beginning of the

sentence.

Paragraph 1, line 4: Please expand the term “placental insufficiency” by including the cause:

excess thromboxane A2. It would be useful to provide information on the specific model used, in

the beginning of the discussion.

To provide information to the readers, the paper discussing the mouse model of placental

insufficiency used in this manuscript was cited at the end of this sentence. 4

Paragraph 5 third line before the end: “Dysregulation of the Wnt pathway been..” should read

"Dyregulation of the Wnt pathway has been...”

Paragraph 7 line 15: “On-gong” should read “ongoing”.

Paragraph 7, line 16: Please replace “MS” with “multiple sclerosis”.

Paragraph 9 line 4: Please remove “of” in “showed less significant of changes”.

Paragraph 9 line 10: This sentence is quite long so please consider removing “including the

corticospinal tract”.

Paragraph 9 last line: There are too many “and” in this sentence. Consider rewriting as follows:

"Samples were composed of pooled tissue from several animals, which minimized biological

variance. Our approach was further validated by qRT-PCR that was highly correlated with RNA Seq findings”.

Paragraph 10: Please remove the part about “suggesting new potential therapeutic targets"

since this paragraph acts as a conclusion, and although there is a lot of detail on the pathways

involved there is no mention of using these pathways for therapeutics anywhere else in the

manuscript.

Reviewer #2

Neonatal WMI is an important problem in the NICU. There are numerous risk factors known -

including IUGR and hyperoxia. Here the authors combine these 2 model systems and perform

RNA-seq to identify gene/pathway changes that may contribute. To my knowledge, this is the

first study that I have seen using this approach in this clinically relevant model system.

The authors explore changes in gene regulation between IUGR and IUGR + hyperoxia to

support a multi-hit hypothesis that infants exposed to multiple insults may be at higher risk for

NDI, particularly in motor outcomes. The authors combined 2 previously characterized models -

thromboxane analogue induced IUGR as well as hyperoxia exposure during periods of white

matter development. The authors then apply RNA-seq techniques to identify changes in genes

and larger genetic pathways in these individual models or when they are combined.

WMI is a significant problem in the NICU - it’s the most common brain injury leading to poor

neurologic outcomes in this population. Moreover, IUGR is an important risk factor for the

development of WMI and as stated by the authors not all IUGR infants develop this injury. For

that reason, the attempt to identify additional factors that influence risk in IUGR infants is both

important and laudable. The results presented here are intriguing and evocative but the n’s are

too small and they don’t have an even distribution of sex - a known determinant in neurologic

outcomes (in humans and animal models). To my knowledge, this work is a new analysis for

IUGR + hyperoxia.

Summary of key findings:

• RNA-seq analysis found small changes in tissue gene expression in all 3 experimental groups.

Interestingly, the greatest change in downregulation of genes controlling WM development and

myelination occurred in the IUGR/Hyperoxia group.

• This study attempts to identify genes/pathways common to all 3 models and identify unique 5

changes to the IUGR/Hyperoxia groups. The data presented support the existence of unique

genetic changes within the IUGR and hyperoxia group.

• Applied IPA highlights genetic pathways that appear to be dysregulated following IUGR and

hyperoxia exposures. Many of these pathways have been previously identified as regulators of

myelination/maturation.

• RNA isolation and QC is standard.

• Analysis used a standard pipeline

• Validation assays used are standard

Major concerns:

1. The changes in gene expression from whole hemisphere analysis is very small. The authors

address this in the discussion and I agree that the impact on oligodendrocyte gene expression

can be diluted with this type of analysis. Given the small Log2 Fold changes observed (<0.5), it

is imperative that the sample number be increased.

For this revision, we performed a second RNA sequencing experiment with an expanded

sample size of individual (non-pooled) samples n =5-8. Additionally, the sample size for the

qRT-PCR validation experiments was also increased in the revision to n = 10-15.

2. When compared to whole hemisphere changes, dissection of WM should exaggerate these

changes in myelination gene expression due to the increased representation of the

oligodendrocyte lineage in the tissue sample. As the authors note in the discussion, this was not

the case. The authors suggest that other white matter regions (cortical or subcortical) may be

more affected. I think it is important to make this determination. As is, this makes us less

confident that the small changes observed in the RNA-seq assay are meaningful.

In the revision myelin gene expression in the subcortical white matter was added.

3. Effects of sex. Generally, the impact of sex on brain injury outcomes have been well documented in both human infants and model systems. Is there sex variation by the exposure

here? The same number of females/males in each of the groups should be used at a minimum.

Ideally, males and females could be pooled and analyzed separately. Pooling of numbers isn’t

consistent. How did they determine how to pool the animals in terms of sex?

The reviewer makes a valid point regarding the impact of sex on brain injury outcomes. To

address this, sexes were separated and compared in our whole hemisphere qRT-PCR analysis.

In regard to pooling, each pooled sample contained a combination of male and female animals.

4. Figure 4D (qPCR X RNA-seq r^2 = 0.75) is barely acceptable.

This figure was removed in the revision to make room for new data.

5. U-46619 does not appear to cross the placenta - based on 11-dehydrothromboxane B2

levels. Can they provide the reference to this data or provide the data itself. This is important

because the authors used U-46619 exposed animals that were >10%ile as controls.

The reference for this data was placed at the of this sentence in the results section. For the

controls, only ethanol and not U-46619 was infused in the pumps.

6. The observed changes in gene expression within tissues could be related to a loss of OL

lineages or a lack of maturation. This is a big question within the field of neonatal WMI - does

the author have any evidence as to which is contributing to the observations presented here?

The reviewer points out an important question. Our data suggests for IUGR with hyperoxia, a

block in maturation given a predominant decrease in DEGs specific to myelinating OLs and

inhibition of regulators known to be involved in OL maturation such as TCF7L2, SOX2, and

BDNF.6

Minor concerns:

1. Labeling in figures would make this easier to read. For example, in Figure 2, labeling heat

maps or volcano plots with IUGR, hyperoxia, and hyperoxia + IUGR.

The heat maps are now labeled in Figure 2.

Bottom line, intriguing and evocative but the n’s are too small + they don’t have an even

distribution of sex which could be a confounder - needs more work. If this were to be published

as is, a thorough discussion on sex factors impacting neurologic outcomes in the discussion

would be important.

In our revision, we increased the sample size for both the RNA Sequencing experiments and

the qRT-PCR validation. To address potential sex differences, sexes were compared in our

qRT-PCR analysis and were addressed in the discussion.

Possible recommendation for future work is to attempt to flow sort oligodendrocyte lineage using

commercially available Olig2-EGFP alleles to isolate cells of interest prior to RNA-seq analysis.

This may assist in identifying changes that are diluted out in the current study.

Our next work uses a genetically modified mouse to isolate RNA specific to oligodendrocytes.

Additionally, this mouse line also further narrows the analysis to mRNA actively being

transcribed
